# Sex-specific Relationship Between Stress Coping Strategies and All-cause Mortality: Japan Multi-Institutional Collaborative Cohort Study

**DOI:** 10.2188/jea.JE20210220

**Published:** 2023-05-05

**Authors:** Mako Nagayoshi, Kenji Takeuchi, Yudai Tamada, Yasufumi Kato, Yoko Kubo, Rieko Okada, Takashi Tamura, Asahi Hishida, Jun Otonari, Hiroaki Ikezaki, Yuichiro Nishida, Chisato Shimanoe, Yuriko N. Koyanagi, Keitaro Matsuo, Haruo Mikami, Miho Kusakabe, Daisaku Nishimoto, Keiichi Shibuya, Sadao Suzuki, Takeshi Nishiyama, Etsuko Ozaki, Isao Watanabe, Kiyonori Kuriki, Naoyuki Takashima, Aya Kadota, Kokichi Arisawa, Sakurako Katsuura-Kamano, Kenji Wakai

**Affiliations:** 1 Department of Preventive Medicine, Nagoya University Graduate School of Medicine, Nagoya, Japan; 2 Department of Psychosomatic Medicine, Kyushu University Graduate School of Medical Sciences, Fukuoka, Japan; 3 Department of Psychosomatic Medicine, International University of Health and Welfare Narita Hospital, Chiba, Japan; 4 Department of Comprehensive General Internal Medicine, Kyushu University Graduate School of Medicine, Faculty of Medical Sciences, Fukuoka, Japan; 5 Department of Preventive Medicine, Faculty of Medicine, Saga University, Saga, Japan; 6 Department of Pharmacy, Saga University Hospital, Saga, Japan; 7 Division of Cancer Information and Control, Aichi Cancer Center Research Institute, Nagoya, Japan; 8 Division of Cancer Epidemiology and Prevention, Aichi Cancer Center Research Institute, Nagoya, Japan; 9 Cancer Prevention Center, Chiba Cancer Center Research Institute, Chiba, Japan; 10 Department of International Island and Community Medicine, Kagoshima University Graduate School of Medical and Dental Sciences, Kagoshima, Japan; 11 School of Health Sciences, Faculty of Medicine, Kagoshima University, Kagoshima, Japan; 12 Department of Emergency and Intensive Care Medicine, Kagoshima University Graduate School of Medical and Dental Sciences, Kagoshima, Japan; 13 Department of Public Health, Nagoya City University Graduate School of Medical Sciences, Nagoya, Japan; 14 Department of Epidemiology for Community Health and Medicine, Kyoto Prefectural University of Medicine, Kyoto, Japan; 15 Laboratory of Public Health, Division of Nutritional Sciences, School of Food and Nutritional Sciences, University of Shizuoka, Shizuoka, Japan; 16 Department of Public Health, Kindai University Faculty of Medicine, Osaka, Japan; 17 Department of Public Health, Shiga University of Medical Science, Shiga, Japan; 18 Center for Epidemiologic Research in Asia, Shiga University of Medical Science, Shiga, Japan; 19 Department of Preventive Medicine, Tokushima University Graduate School of Biomedical Sciences, Tokushima, Japan

**Keywords:** sex differences, stress coping strategies, perceived stress, all-cause mortality, Japan

## Abstract

**Background:**

Stress coping strategies are related to health outcomes. However, there is no clear evidence for sex differences between stress-coping strategies and mortality. We investigated the relationship between all-cause mortality and stress-coping strategies, focusing on sex differences among Japanese adults.

**Methods:**

A total of 79,580 individuals aged 35–69 years participated in the Japan Multi-Institutional Collaborative Cohort Study between 2004 and 2014 and were followed up for mortality. The frequency of use of the five coping strategies was assessed using a questionnaire. Sex-specific, multivariable-adjusted hazard ratios (HRs) for using each coping strategy (“sometimes,” and “often/very often” use versus “very few” use) were computed for all-cause mortality. Furthermore, relationships were analyzed in specific follow-up periods when the proportion assumption was violated.

**Results:**

During the follow-up (median: 8.5 years), 1,861 mortalities were recorded. In women, three coping strategies were related to lower total mortality. The HRs for “sometimes” were 0.81 (95% confidence interval [CI], 0.67–0.97) for emotional expression, 0.79 (95% CI, 0.66–0.95) for emotional support-seeking, and 0.80 (95% CI, 0.66–0.98) for disengagement. Men who “sometimes” used emotional expression and sometimes or often used problem-solving and positive reappraisal had a 15–41% lower HRs for all-cause mortality. However, those relationships were dependent on the follow-up period. There was evidence that sex modified the relationships between emotional support-seeking and all-cause mortality (*P* for interaction = 0.03).

**Conclusion:**

In a large Japanese sample, selected coping strategies were associated with all-cause mortality. The relationship of emotional support-seeking was different between men and women.

## INTRODUCTION

Coping strategies are cognitive and behavioral reactions to stressful events or situations.^1^ It is known that the impact of psychological stress on health is influenced by how we cope with stress by regulating emotions, situations, or behaviors.^2^ Previous studies have suggested that certain coping strategies are associated with higher risks of various health outcomes, such as depression^3^ and cardiovascular diseases.^4^^,^^5^ Adaptive coping strategies have been associated with lower risks.^6^ Although stress responses may not always be categorized as adaptive/maladaptive coping strategies due to the situational dependence of the various coping strategies used,^7^ coping dispositions could be linked to individual health.

These strategies should differ by sex,^8^ as various backgrounds influence perceived stress. Cultural and social environments have been different among men and women (eg, life events, gender role norms at home, and job position and/or salary gaps^9^^–^^11^), which may partially contribute to the higher suicide rate in the former^12^ and higher poverty rate in the latter.^10^ Biological sex differences (eg, dramatic/gradual changes in hormonal balance according to age and cardiovascular and neuroendocrine responses to stress^13^) have also led to sex differences in stress reactions. Previous studies have shown that women are more likely to perceive stress^14^ and experience stress-induced physical and mental symptoms. They have various coping strategies and use them more frequently than men.^8^^,^^14^ Women reportedly tend to focus on emotional coping^14^^,^^15^ and mobilize more social support during periods of stress.^8^^,^^16^ In an early report by Carver et al in 1989,^7^ they mentioned that, of the sex difference in the results, “the largest and most reliable of these differences were on tendencies (in women) to focus on and vent emotions, and seek social support, both for instrumental and emotional reasons,” and they were “consistent with sex role stereotypes.”^7^ Generally, men prefer coping styles that can be practiced by themselves rather than relying on others. This could be why men rarely share their feelings under stress,^14^ and are more likely to use alcohol, tobacco, or drugs than women.^7^ It is known that psychosocial stress increases the risk of mortality not only through biological changes (eg, alteration of the autonomic nervous system, endocrine, and immune systems^17^^,^^18^) but also maladaptive behavioral changes, as they contribute to hypertension, dyslipidemia, diabetes, and arteriosclerosis and result in the development of cardiovascular diseases and cancer. An existing study indicated sex differences in the relationship pathways between coping strategy and all-cause mortality.^19^

Therefore, understanding the sex-specific relationships of coping strategies to all-cause mortality is important to clarify how to intervene in the target population depending on their attributes. However, many studies have not focused on sex differences in the relationship between coping strategies and all-cause mortality. Sex-specific evidence from general populations, rather than patients, is also insufficient. Previous studies often focused on one specific coping strategy, such as emotional expression,^4^^,^^19^^–^^24^ and sex and coping strategies-combined risks.^25^^,^^26^ Moreover, the perceived stress level has not been accounted for in these relationships. Therefore, we conducted a large longitudinal study in Japan to test the following hypotheses: a) specific coping strategies may be related to a lower risk of all-cause mortality, even after accounting for perceived stress level; and b) the relationship between coping strategies and all-cause mortality would differ by sex.

## METHODS

### Design/setting and participants

We targeted 92,560 men and women aged 35–69 years who participated in the Japan Multi-Institutional Collaborative Cohort (J-MICC) Study between 2004 and 2014.^27^^,^^28^ The J-MICC study aimed to examine gene-environment interactions in lifestyle-related diseases and mortality among Japanese.^27^ The detailed information of the J-MICC study has been described elsewhere.^27^^,^^28^

**Figure 1.**
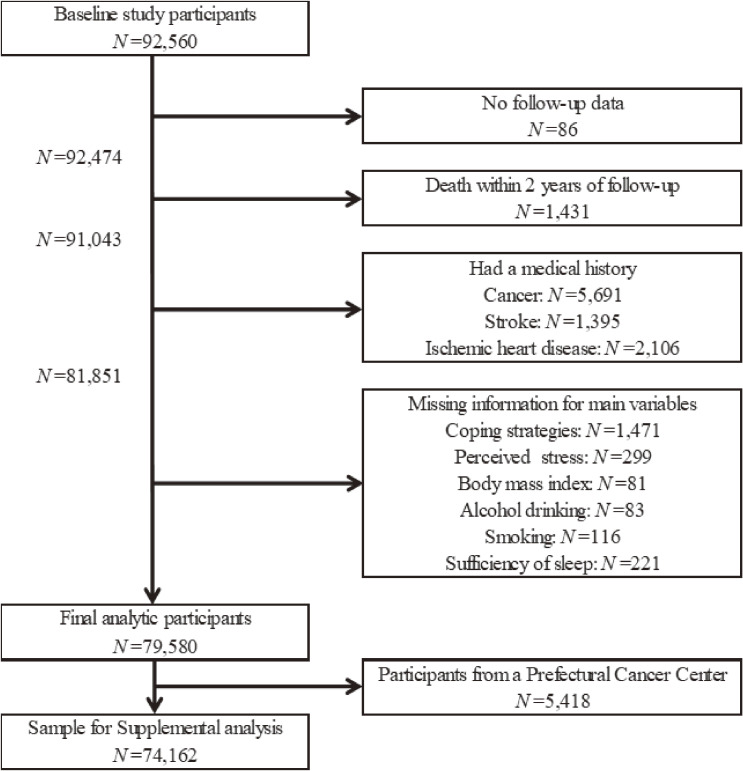
Study sample flow chart

The flow chart of the study sample is shown in Figure 1. Participants without follow-up data (*n* = 86) or mortality within 2 years of follow-up (*n* = 1,431) were excluded from the analyses. We also excluded participants who had a history of cancer (*n* = 5,691), stroke (*n* = 1,395), or ischemic heart disease (*n* = 2,106) at baseline, and with missing data on the main interest, including coping strategies (*n* = 1,471), perceived stress (*n* = 299), body mass index (BMI) (*n* = 81), alcohol drinking (*n* = 83), smoking (*n* = 116), or sleep sufficiency (*n* = 221). Our final analytic sample included 79,580 participants (34,087 men and 45,493 women). The study protocol was approved by the ethics committees of the Nagoya University Graduate School of Medicine, and each principal institute conducted the study at each site. Written informed consent was obtained from all participants. All analyses in this study were based on the dataset “ver.20200819” provided by the central office of the J-MICC Study.

### All-cause mortality

Information on participants’ mortalities was collected from the baseline visit (2004–2014) until the end of 2016 or 2017, except for one area where the information was obtained on December 31, 2012, 2013, and 2016. Participants who moved out of the study region were censored from the follow-up when they left.

### Coping strategies and perceived stress

Five coping strategies selected from a dispositional version of the General Coping Questionnaire^29^^,^^30^ or the brief COPE^31^ were assessed using the question: “How do you cope with various problems and unfavorable events in daily life?” Participants were requested to answer the frequency of each coping strategy with four choices: “very few,” “sometimes,” “often,” or “very often.” Due to the limited “very often” responses, “often” and “very often” responses were grouped into one category (“often/very often”) for analysis in this study. The coping strategies were these: (1) “I express my negative feelings and thoughts” (emotion expression); (2) “I consult with someone close and ask them for encouragement” (emotional support-seeking); (3) “I try to interpret the problem in a favorable way” (positive reappraisal); (4) “I try hard to solve the problem (problem-solving); and (5) “I let the problem take its course” (disengagement).^32^

Perceived stress and coping strategies were evaluated using a self-reporting questionnaire. The level of perceived stress during the past year was assessed by this question: “How much stress did you experience in the last year?”^32^^,^^33^ The answer had four choices: “I felt significantly stressed,” “I felt somewhat stressed,” “I felt a little stress,” and “I felt no stress at all.”

### Covariates

Covariates were assessed at the baseline examination (2004–2014). Information on age, sex, socioeconomic status, smoking, alcohol use, physical activity, sleep, medical history, and family history of cancer was obtained using self-administered questionnaires. Trained interviewers checked the responses. The socioeconomic status included educational attainment (high school graduate or less, beyond high school, missing). Smoking status was divided into four groups: never, former, current (<20 or ≥20 cigarettes per day). Detailed information on physical activity has been described elsewhere.^34^^,^^35^ The physical activity during leisure time was estimated using a method similar to the International Physical Activity Questionnaire.^36^ The amount of daily physical activity and leisure time was estimated as metabolic equivalent (MET)-h/week (MET level × hours of activity per week), respectively. Self-reported sufficiency of sleep was obtained using the question, “Do you think your daily sleep is sufficient for you?” The choices were “enough,” “somewhat lacking,” ‘lacking,’ and “unknown.” We categorized them into two groups by responses: “enough,” or not. We used this index to assess participants’ sleep instead of sleep duration because the latter was strongly dependent on age. Medical history included hypertension and diabetes, and was categorized as yes or not. Family history of cancer was detected through the medical history of their parents (yes, no, unknown, or missing). Weight (kg) and height (cm) were measured in light clothes during the first visit at baseline. Self-reported values were used if weight and height were not measured. BMI was calculated as weight (kg) divided by height (m) squared.

### Statistical analyses

Descriptive statistics were generated for all-cause mortality-related variables and covariates, including perceived stress and coping strategies, stratified by sex. Analysis of variance and χ^2^ tests were used to examine differences in means for continuous variables or proportions for categorical variables. Spearman correlation coefficients (*r*_s_) were calculated for key variables of interest, which included the frequency of each coping strategy and the magnitude of perceived stress as a continuous variable (0–3). Cox proportional hazards regression models were used to estimate hazard ratios (HRs) and 95% confidence intervals (CIs) for the relationship between each coping strategy and all-cause mortality. The HRs were computed for “sometimes,” and “often/very often” use of each coping strategy (versus “very few” use). The relationship between each coping strategy and all-cause mortality was examined using Cox proportional hazards models. The follow-up time was calculated from the date of the baseline visit until mortality, the date of moveout, or the end of follow-up, whichever came first.

We constructed and compared a series of nested models: model 1 was adjusted for age as a covariate, and model 2 was additionally adjusted for socioeconomic status (educational attainment), physical risk factors (BMI, history of hypertension, and diabetes), combined genetic and environmental factors (father’s and mother’s history of cancer), and behavioral risk factors (smoking status, alcohol use, physical activity in daily life and leisure time, sufficiency of sleep), with perceived stress as a psychological factor. The proportional hazards assumption was evaluated by testing the interaction between coping strategy categories and the natural log of follow-up time, and visual inspection of graphs of the survival function versus time, stratified by the frequency of coping strategies. When the proportionality assumption was violated, the follow-up period was divided into two phases at the median point until the proportionality was confirmed. For women, the follow-up period for “problem-solving” was divided into two phases at the median of 8.5 years. For men, the median of 8.5 years and the 25th percentile of 5.5 years was used to divide “emotion expression,” “positive reappraisal” and “problem-solving.” Each coping strategy scale was implemented separately based on Carver’s recommendation, who created the Brief COPE.^31^

For sensitivity analyses, as participants from a clinical setting could have different distributions in psychological stress and coping strategies for the possible worse health status and the higher mortality rate, we excluded 5,418 participants from one large prefectural cancer center, which had 18.0% (*n* = 335) of the total mortalities. Effect modifications of the associations between coping strategies and mortality by sex and confounding factors were assessed by including cross-product terms of each confounding factor and coping strategies in model 2. All analyses were performed using SAS (version 9.4; SAS Institute Inc., Cary, NC, USA). All *P*-values reported were two-tailed, and values of less than 0.05 were considered statistically significant.

## RESULTS

**Figure 2.**
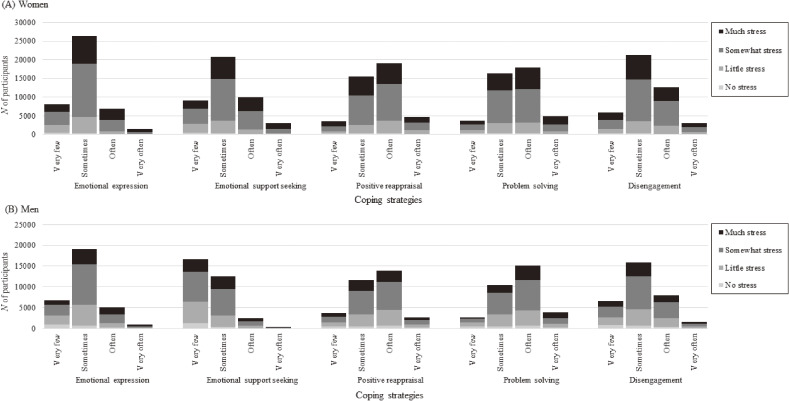
Distribution of study participants according to frequency of each coping strategy and perceived stress in women (*A*) and men (*B*): the J-MICC Study. The distribution of participants in each coping strategy was similar in women and men; the majority of participants distributed in “sometimes” and/or “often” in each coping strategies, other than emotional support-seeking in men.

Table 1 shows the sex-specific characteristics of the study participants. The final analytic sample included 79,580 participants (57.2% female; average age, 54.7 years; mean BMI, 23.0 kg/m^2^). Significant stress was reported by 27.1% of participants, and the proportion was higher in women (Table 1). The sex-specific distribution of study participants according to the frequency of each coping strategy and perceived stress level are shown in Figure 2. Distributions of participants in the frequency of coping strategy and perceived stress level were similar between sexes, except for emotional support-seeking behavior; the majority (78.5%) of women answered “sometimes” or more frequently, whereas the majority (52.3%) of men answered “very few.” About 60% of the men and women answered “sometimes” for emotional expression, and about 50% of them answered “sometimes” for disengagement. For positive reappraisal and problem-solving, the majority of both sexes answered “often” (Figure 2).

**Table 1.** Sex-stratified characteristics of study participants: the J-MICC Study 2004–2014

	Women	Men
	*N* = 45,493	*N* = 34,087
**Demographics**		
Age, years^a^	54.3 (9.4)	54.3 (9.4)
Education, %		
≤High School Graduate	39.9	37.9
>High School Graduate	36.4	41.4
Missing	23.7	20.8
**Behavioral characteristics**		
Physical activities, METs/week^a^		
Total	77.8 (83.9)	84.7 (96.7)
Daily	25.5 (13.8)	24.1 (15.9)
Leisure time	52.4 (82.0)	60.5 (94.9)
Cigarette smoking, %		
Never	85.7	29.7
Former	7.2	39.8
<20 cigarettes per day	4.7	9.5
≥20 cigarettes per day	2.4	21.0
Current alcohol drinker, %		
Current	37.6	76.8
Former	1.8	3.0
Never	60.6	20.2
**Physiologic characteristics**		
Body mass index, kg/m^2 a^	22.3 (3.3)	22.3 (3.3)
Prevalent diabetes, %	3.1	3.3
Prevalent hypertension, %	14.8	15.4
Having a very satisfied sleep	47.5	47.5
**Genetic and environmental factors**		
Mother’s history of cancer, %	18.7	16.9
Father’s history of cancer, %	27.1	26.4
**Perceived stress level, %**		
Much stress	31.1	21.6
Somewhat stress	49.4	46.2
Little stress	16.9	26.4
No stress	2.5	5.8
**Coping strategies**		
Emotional expression		
Very often	3.6	3.2
Often	16.0	15.9
Sometimes	61.6	59.9
Very few	18.8	20.9
Missing	0.1	0.1
Emotional support-seeking		
Very often	6.8	1.2
Often	22.8	7.9
Sometimes	48.9	38.6
Very few	21.5	52.3
Missing	0.1	0.2
Positive reappraisal		
Very often	11.0	8.4
Often	44.6	43.4
Sometimes	36.3	36.7
Very few	8.1	11.4
Missing	0.2	0.2
Problem solving		
Very often	11.2	11.9
Often	41.9	46.9
Sometimes	38.4	32.7
Very few	8.6	8.5
Missing	0.2	0.2
Disengagement		
Very often	6.8	4.7
Often	29.7	24.8
Sometimes	49.6	49.8
Very few	13.9	20.7
Missing	0.3	0.3

J-MICC Study, Japan Multi-institutional Collaborative Cohort Study.

^a^Means (standard deviations).

During the median follow-up of 8.5 years, 1,861 mortalities were observed, with crude total mortality of 2.9/1,000 person-years. Perceived stress level was negatively correlated with age (men: *r*_s_ = −0.31; women: *r*_s_ = −0.21) (Table 2). Most of the frequency of using coping strategies was also negatively correlated with age in men and women and positively correlated with each other except for problem-solving and disengagement, which were negatively correlated in men and women (*r*_s_ = −0.18 and −0.17, respectively) (Table 2). The strongest correlations of coping strategies were positive reappraisal and problem-solving in both sexes (*r*_s_ = 0.51, and 0.52, respectively) (Table 2).

**Table 2.** Correlation between perceived stress and coping strategies at baseline: the J-MICC Study 2004–2014

		Women						Men					
	
		1	2	3	4	5	6	1	2	3	4	5	6
1	Age	—	—	—	—	—		—	—	—	—	—	—
2	Perceived stress level^a^	−0.21^b^	—	—	—	—		−0.31^b^	—	—	—	—	—
3	Emotional expression^a^	−0.15^b^	0.19^b^	—	—	—		−0.07^b^	0.19^b^	—	—	—	—
4	Emotional support-seeking^a^	−0.24^b^	0.18^b^	0.27^b^	—	—		−0.18^b^	0.16^b^	0.19^b^	—	—	—
5	Positive reappraisal^a^	−0.01	−0.02^b^	0.05^b^	0.20^b^	—		−0.05^b^	0.01	0.07^b^	0.17^b^	—	—
6	Problem solving^a^	−0.07^b^	0.11^b^	0.10^b^	0.24^b^	0.52^b^		−0.10^b^	0.14^b^	0.14^b^	0.19^b^	0.51^b^	—
7	Disengagement^a^	−0.10^b^	0.01	0.06^b^	0.02^b^	0.06^b^	−0.17^b^	−0.10^b^	0.05^b^	0.04^b^	0.00	0.02	−0.18^b^

J-MICC Study, Japan Multi-institutional Collaborative Cohort Study.

^a^Numbers are Spearman correlation coefficients calculated by the frequency of each coping strategy and the magnitude of stress as a continuous variable (0–3).

^b^Correlation is significant at the 0.001 level (two-tailed).

### Coping strategies and all-cause mortality

#### Emotional expression

After adjusting for demographics, health behaviors, physiological and psychological factors, and genetic and environmental factors, we found that women who answered “sometimes” for emotional expression had a lower HR of all-cause mortality than those of “very few.” The HR was 0.81 (95% CI, 0.67–0.97) (model 2 in Table 3). In men, the relationship between the frequency of emotional expression and all-cause mortality was dependent on the follow-up duration. Men used emotional expression “sometimes” had a lower HR for all-cause mortality in the follow-up period of 5.5–8.5 years (HR 0.77; 95% CI, 0.60–0.98) (model 2 in Table 4). The relationships in men differed according to age at the median of 8.5 years or longer follow-up period (*P* for interaction = 0.03) (data not shown). When stratified by age group (<60 years or ≥60 years), younger men used emotional expression “sometimes” and “often/very often had higher all-cause mortality. The respective HRs were 1.90 (95% CI, 0.96–3.74) and 2.02 (95% CI, 0.93–4.35). There was no statistical evidence to support that sex modified the relationships between emotional expression and all-cause mortality during the entire follow-up period (*P* for interaction = 0.77).

**Table 3.** Relationship of coping strategies to all-cause mortality in women: the J-MICC Study 2004–2014

Coping strategies^a^	*N* total mortality	Person years	*N* participants	Model 1		Model 2	
				HR (95% CI)		HR (95% CI)	
	
				Follow-up period^b^		Follow-up period^b^	
				<8.5 years	≥8.5 years	<8.5 years	≥8.5 years
Emotional expression							
Very few	162	70,162	8,548	Reference		Reference	
Sometimes	374	230,964	27,988	**0.80 (0.66–0.96)**		**0.81 (0.67–0.97)**	
Often/very often	109	72,233	8,919	0.84 (0.66–1.08)		0.84 (0.65–1.08)	

Emotional support-seeking							
Very few	204	81,272	9,758	Reference		Reference	
Sometimes	288	183,622	22,219	**0.77 (0.64–0.92)**		**0.79 (0.66–0.95)**	
Often/very often	152	108,319	13,459	0.85 (0.68–1.05)		0.87 (0.70–1.08)	

Positive reappraisal							
Very few	63	29,443	3,662	Reference		Reference	
Sometimes	223	134,575	16,474	0.86 (0.65–1.13)		0.89 (0.67–1.18)	
Often/very often	357	208,960	25,271	0.81 (0.64–1.04)		0.89 (0.68–1.17)	

Problem solving							
Very few	77	32,643	3,906	Reference^c^	Reference^d^	Reference^c^	Reference^d^
Sometimes	257	142,631	17,418	0.88 (0.66–1.18)^c^	1.01 (0.61–1.69)^d^	0.95 (0.71–1.28)^c^	1.06 (0.63–1.77)^d^
Often/very often	308	197,664	24,080	0.81 (0.61–1.08)^c^	0.80 (0.48–1.32)^d^	0.92 (0.69–1.24)^c^	0.81 (0.49–1.36)^d^

Disengagement							
Very few	120	52,513	6,314	Reference		Reference	
Sometimes	283	185,588	22,504	**0.78 (0.63–0.97)**		**0.80 (0.64–0.99)**	
Often/very often	239	134,501	16,548	0.95 (0.76–1.18)		0.95 (0.76–1.19)	

CI, confidence interval; HR, hazard ratio; J-MICC Study, Japan Multi-institutional Collaborative Cohort Study.

^a^Coping strategies were separately included in the models.

^b^Follow-up period was up to 13.9 years. When the proportionality assumption was violated, follow-up period was divided into two phases at the median of 8.5 years.

^c^Follow-up period was <8.5 years (the 0–50^th^ percentile of the total follow-up period).

^d^Follow-up period was ≥8.5 years (the 50–100^th^ percentile of the total follow-up period).

Model 1: Adjusted for age.

Model 2: Adjusted for model 1 + socioeconomic status (educational attainment), physical factors (body mass index, diabetes, hypertension), genetic and environmental factors (father and mother’s histories of cancer), behavioral risk factors (smoking status, alcohol drinking, physical activity in daily and in leisure time, sufficiency of sleep), perceived stress level.

Results marked in **bold** indicate a significant association with all-cause mortality (*P* < 0.05).

**Table 4.** Relationship of coping strategies to all-cause mortality in men: the J-MICC Study 2004–2014

Coping strategies^a^	*N* total mortality	Person years	*N* participants	Model 1			Model 2		
				HR (95% CI)			HR (95% CI)		
	
				Follow-up period^b^			Follow-up period^b^		
				<5.5 years	5.5–8.5 years	≥8.5 years	<5.5 years	5.5–8.5 years	≥8.5 years
Emotional expression									
Very few	303	56,177	7,131	Reference^c^	Reference^d^	Reference^e^	Reference^c^	Reference^d^	Reference^e^
Sometimes	708	164,935	20,411	0.96 (0.79–1.18)^c^	**0.69 (0.55–0.88)** ^d^	0.94 (0.71–1.24)^e^	0.95 (0.78–1.17)^c^	**0.77 (0.60–0.98)** ^d^	0.97 (0.72–1.29)^e^
Often/Very often	203	52,644	6,510	0.86 (0.65–1.13)^c^	**0.72 (0.52–0.98)** ^d^	0.97 (0.68–1.37)^e^	0.76 (0.57–1.01)^c^	0.84 (0.61–1.16)^d^	0.98 (0.69–1.41)^e^

Emotional support-seeking									
Very few	702	144,413	17,810	Reference			Reference		
Sometimes	424	104,705	13,131	1.04 (0.92–1.18)			1.07 (0.95–1.21)		
Often/Very often	88	24,477	3,088	1.00 (0.80–1.24)			1.06 (0.84–1.32)		

Positive reappraisal									
Very few	189	30,841	3,889	Reference^c^	Reference^d^	Reference^e^	Reference^c^	Reference^d^	Reference^e^
Sometimes	425	100,362	12,484	0.84 (0.64–1.09)^c^	0.83 (0.61–1.13)^d^	**0.61 (0.43–0.84)** ^e^	0.78 (0.60–1.02)^c^	0.86 (0.62–1.17)^d^	**0.63 (0.45–0.89)** ^e^
Often/Very often	597	142,366	17,654	0.93 (0.72–1.20)^c^	**0.69 (0.51–0.93)** ^d^	**0.67 (0.49–0.92)** ^e^	0.92 (0.72–1.19)^c^	0.76 (0.56–1.03)^d^	**0.71 (0.52–0.98)** ^e^

Problem solving									
Very few	157	23,332	2,889	Reference^c^	Reference^d^	Reference^e^	Reference^c^	Reference^d^	Reference^e^
Sometimes	397	89,791	11,121	0.93 (0.70–1.25)^c^	**0.58 (0.42–0.78)** ^d^	1.12 (0.75–1.68)^e^	0.92 (0.69–1.24)^c^	**0.63 (0.46–0.87)** ^d^	1.20 (0.80–1.81)^e^
Often/Very often	659	160,476	20,023	0.99 (0.76–1.31)^c^	**0.54 (0.40–0.71)** ^d^	1.07 (0.73–1.58)^e^	1.02 (0.77–1.34)^c^	**0.59 (0.44–0.80)** ^d^	1.16 (0.78–1.72)^e^

Disengagement									
Very few	301	57,408	7,035	Reference			Reference		
Sometimes	587	136,523	16,937	0.96 (0.83–1.10)			0.99 (0.86–1.14)		
Often/Very often	324	79,414	10,027	0.94 (0.80–1.10)			0.95 (0.81–1.12)		

CI, confidence interval; HR, hazard ratio; J-MICC Study, Japan Multi-institutional Collaborative Cohort Study.

^a^Coping strategies were separately included in the models.

^b^Follow-up period was up to 13.9 years. When the proportionality assumption was violated, follow-up period was divided into two phases at the median point until the proportionality was confirmed.

^c^Follow-up period was <5.5 years (the 0–25^th^ percentile of the total follow-up period).

^d^Follow-up period was 5.5–8.5 years (the 25–50^th^ percentile of the total follow-up period).

^e^Follow-up period was >8.5 years (the 50–100^th^ percentile of the total follow-up period).

Model 1: Adjusted for age.

Model 2: Adjusted for model 1 + socioeconomic status (educational attainment), physical factors (body mass index, diabetes, hypertension), genetic and environmental factors (father and mother’s histories of cancer), behavioral risk factors (smoking status, alcohol drinking, physical activity in daily and in leisure time, sufficiency of sleep), perceived stress level.

Results marked in **bold** indicate a significant association with all-cause mortality (*P* < 0.05).

#### Emotional support-seeking

Compared with participants who answered “very few” for emotional support-seeking, the HR of all-cause mortality among women who answered “sometimes” was 0.79 (95% CI, 0.66–0.95) after full adjustment for covariates (model 2 in Table 3). Emotional support-seeking was not related to all-cause mortality in men (model 2 in Table 4). We found evidence that sex modified the relationships between emotional support-seeking and all-cause mortality (*P* for interaction = 0.03).

#### Positive reappraisal

Among men, the relationship between the frequency of positive reappraisal and all-cause mortality was dependent on the follow-up duration. Although men who answered “sometimes” and “often/very often” for positive reappraisal tended to have lower hazard ratios of all-cause mortality than those of “very few,” throughout the follow-up period, the relationships were most prominent in the 8.5 years or longer follow-up period (model 2 in Table 4). The respective HRs were 0.63 (95% CI, 0.45–0.89) and 0.71 (95% CI, 0.52–0.98). The relationships differed according to smoking status at the 5.5 years follow-up period in men (*P* for interaction = 0.03) (data not shown). The frequent positive reappraisal in men who currently smoked less than 20 cigarettes per day had a relatively high risk of all-cause mortality; HR was 2.02 (95% CI, 0.97–4.24) for “often/very often.” Positive reappraisal was not related to all-cause mortality in women (model 2 in Table 3). There was no statistical evidence suggesting that sex modified the relationships (*P* for interaction = 0.70).

#### Problem-solving

The relationship between the frequency of problem-solving and all-cause mortality was dependent on the follow-up duration in men and women. Men who answered “sometimes” or more often for problem-solving had lower HRs for all-cause mortality than those of “very few” (model 2 in Table 4) in the 5.5–8.5 years follow-up period. The respective HRs were 0.63 (95% CI, 0.46–0.87) for “sometimes,” and 0.59 (95% CI, 0.44–0.80) for “often/very often.” No association was observed for all-cause mortality among women (model 2 in Table 3). There was no statistical evidence that sex modified the relationships (*P* for interaction = 0.97).

#### Disengagement attitude

Women who answered “sometimes” for disengagement attitude had a lower risk of all-cause mortality (HR 0.80; 95% CI, 0.64–0.99) than “very few” (model 2 in Table 3). Disengagement attitudes were not related to all-cause mortality in any follow-up periods in men. There was no statistical evidence that sex modified the relationships (*P* for interaction = 0.59).

### Interaction between perceived stress and coping strategies regarding all-cause mortality

There was no evidence that perceived stress modified the relationship between coping strategies and mortality.

### Sensitivity analyses

As participants who were recruited in a clinical setting could have worse health status and a higher mortality rate, additional analyses excluding participants from a large prefectural cancer center (*n* = 5,418, 6.8% of the total sample) were conducted (eTable 1 and eTable 2). The exclusion reduced 18.0% (*n* = 335) of total mortalities. However, the relationships between coping strategies and all-cause mortality did not appreciably change when we excluded participants from the prefectural cancer center.

## DISCUSSION

We found that stress coping strategies were related to a lower risk of all-cause mortality in 79,580 healthy individuals in Japan. Notably, the relationships between coping strategies and mortality were independent of perceived stress levels. Further, there was a sex-specific statistical difference in all-cause mortality related to seeking emotional support; emotional support-seeking was related to a 21% lower risk of mortality in women, but not men. To the best of our knowledge, this is the first study assessing sex-specific relationships between coping strategies and all-cause mortality, accounting for perceived stress levels among the healthy population.

Evidence for emotional expression/suppression is relatively large,^4^^,^^19^^–^^24^ as it has been known as an important coping strategy. Anger expression may be the most essential figure of emotional expression.^4^^,^^19^^,^^21^^–^^23^ Sex-combined results or those only for women have shown that suppression of emotion or anger was related to mortality in Western countries.^4^^,^^20^^–^^23^^,^^37^ Chapman et al^20^ reported that the 75th percentile on the emotional suppression score had a 35% higher risk of all-cause mortality than the lowest (25^th^) percentile score during 12 years of follow-up in a nationally representative sample in the United States. Harburg et al^19^ assessed participants’ responses to hypothetical unfair anger-provoking situations in an American community. They showed that higher scores for suppressing anger were associated with all-cause mortality during 17 years of follow-up. Stürmer et al^23^ reported that there was no relationship between the composite outcome of cancer incidence and mortality in an 8.5-year follow-up of 5,114 participants in Germany. Hirokawa et al^24^ assessed sex-specific relationships of rationality/anti-emotionality (R/A) personality and all-cause mortality in a 7-year follow-up study of 36,990 Japanese residents. They reported that personality traits were related to a lower risk of mortality in women; those with a middle and higher R/A personality had a 17–18% lower risk of all-cause mortality than the low score group. Our results support the evidence that emotional expression may be beneficial in preventing all-cause mortality in men and women. In this study, the “sometimes” use of emotional expression was related to a lower risk of all-cause mortality, suggesting that this coping strategy benefitted the majority of men and women (about 60%). The high proportion of people using emotional expression “sometimes” might have been partially due to the cultural background in Japan and response tendencies unique to Japanese. In Japanese culture, people consider “expression of anger” shameful^38^ and tend to avoid extreme answers.^39^^,^^40^ The pathway of the relationships could differ by sex, as Harburg et al pointed out.^19^ The impact of men’s suppressed anger on all-cause mortality interacted significantly with systolic blood pressure and bronchial problems, whereas women showed more direct relationships with mortality. It is unclear why younger men in this population who used emotional expression reported higher all-cause mortality risk during the longer follow-up period. Perhaps the sex-specific pathways might have influenced this relationship. Future studies should confirm the sex differences regarding mortality, including the aforementioned pathways.

Evidence for the relationship between other coping strategies and all-cause mortality is relatively limited,^25^^,^^41^ but recent sex-combined results^25^ and a systematic review and meta-analysis^26^ suggest that the relationships would be significant. We have added sex-specific evidence to the relationships. The most significant sex difference in our participants was distribution and relationships to mortality regarding emotional support-seeking; the majority of women (78.5%) sought it frequently, while the majority of men (52.3%) rarely sought such kind of support. Emotional support-seeking was beneficial only for women (*P* for interaction = 0.03); the current study found a 19% lower risk of all-cause mortality for women seeking emotional support. No other population-based study has revealed a sex-specific relationship between emotional support-seeking as a coping strategy and mortality. Previous studies have reported that women tend to focus on emotional coping^14^^,^^15^ and seek social support.^7^^,^^8^^,^^16^

Although men who used positive reappraisal sometimes or more often reported lower all-cause mortality risk throughout the follow-up period, our data suggest that this coping strategy might most effectively prevent long-term mortality in men. Svensson et al also reported that positive reappraisal was related to a 17% lower sex-combined risk of cancer mortality^25^ and 37% lower risk of ischemic heart disease mortality^41^ in more than 55,000 healthy Japanese middle-aged subjects. As there was no evidence for women in this study, this type of coping would be important to prevent mortality only in men.

Men who sometimes or more often used problem-solving had a 37–41% lower risk of all-cause mortality in the follow-up period of 5.5–8.5 years in the present study. Further studies should clarify the cause of this relationship, including follow-up duration or life-event consequences, in men. No significant relationship was observed in women. Although no similar finding has been reported to date, this coping behavior could be a burden for women in some cases. Sasaki et al^29^ reported sex differences in the association between problem-solving and burnout in 1,291 Japanese nurses. The frequent use of problem-solving predicted high professional efficacy in both sexes, whereas there was concurrent high exhaustion only in women.

We found that the “sometimes” use of disengagement attitude was related to a 20% lower risk of all-cause mortality only in women. It has been considered as an avoidance coping strategy^42^ and, conversely, has been associated with maladaptive or poor outcomes. However, it could help in accepting and adapting situations related to stress.^43^ Our results could be understood based on Folkman’s claim that “believing that an event is controllable does not always lead to a reduction in stress or to a positive outcome, and believing that an event is uncontrollable does not always lead to an increase in stress or a negative outcome.”^44^ However, disengagement attitudes in men may not substantially be related to all-cause mortality. Throughout the current study analysis, we did not find any evidence to support that perceived stress level modified the relationships between coping strategies and mortality. Previous studies neglect stress level roles in the relationship between coping strategies and mortality, even though stress level could influence individuals’ coping strategies. Our results suggest that coping strategies would be a beneficial marker for preventing mortality, which is independent of their perceived stress level. As suggested in this and previous studies,^45^ it is important to take into account the differences in their social and behavioral backgrounds while assessing the relationships between coping strategies and mortality. Sex differences may have the most significant influence on these backgrounds. Moreover, they could reflect differences in their cultural and social backgrounds. Since personality^22^^,^^46^ and genetic constitutions^47^ have been shown to partially explain stress perception and coping strategies, analyses that include these factors will help clarify how to intervene depending on the attributes of the target population.

This study has several limitations. First, although coping strategies were abstracted by earlier J-MICC study investigators partly from validated questionnaires, a dispositional version of the General Coping Questionnaire^29^^,^^30^ or the brief COPE,^31^ they were not validated. Nonetheless, this five-item coping strategy is a useful tool to assess people’s stress coping styles and is associated with disease-related biological indicators.^32^^,^^48^ Moreover, this five-item scale enabled us to ask five components of coping strategies in a large cohort with less burden to the participants compared to using the original 32-item GCQ^30^ or the brief COPE with 28 items.^31^ Second, we used a single-item measure of perceived stress. Although assessments with single-item measures have been reported as reliable at measuring stress with validity similar to longer questionnaires,^49^ we could not estimate the types of stress (eg, life events or job stress). Moreover, as this self-reported measure was administered at a single point, which was a median of 8 years before mortality, the length of the stress (eg, for days or years; temporal or chronic stress) was unknown. Future studies with repeated measurements to detect stress types may be needed to assess the influence of long-term or chronic stress on mortality. Third, as the baseline survey of this study was conducted between 2004 and 2014, the range of follow-up duration was relatively wide. Since proportional assumption tests suggest the dependence of follow-up duration for some relationships among men, further studies will be needed to assess whether the impact of such coping strategies in men is time-dependent or influenced by a life-event. Sex-specific relationships and those differences of proportionalities will need to be confirmed by future studies. Last, although we adjusted for potential confounders, unmeasured confounders (eg, depression, social network, and social support) could have influenced the relationship between stress coping strategies and all-cause mortality^50^ through other pathways. Despite these limitations, our study had several strengths. The J-MICC study has a large sample size with a wealth of information on potential confounding factors. They allowed us to examine sex-specific relationships between each frequency of using stress coping strategies and all-cause mortality, and whether they were independent of perceived stress and known risk factors. Moreover, systematically collected information on participants’ mortality and its confirmation using a standardized protocol enabled us to complete their follow-up.

### Conclusion

Our results showed that emotional expression, emotional support-seeking, and disengagement attitude were associated with lower mortality in women, whereas emotional expression, positive reappraisal, and problem-solving were associated with lower mortality in men. Our results on the interaction between the emotional support-seeking and sex highlight the importance of exploring sex differences to clarify how to intervene depending on the attributes of the target population.

## SUPPLEMENTARY MATERIAL

eTable 1. Relationship of coping strategies to all-cause mortality in women after exclusion of participants from a prefectural cancer center: the J-MICC Study, 2004–2014

eTable 2. Relationship of coping strategies to all-cause mortality in men after exclusion of participants from a prefectural cancer center: the J-MICC Study, 2004–2014
