# Supplementary material for: Sex-specific Relationship Between Stress Coping Strategies and All-cause Mortality: Japan Multi-Institutional Collaborative Cohort Study
Source: J Epidemiol. 2023 May 5;33(5):236–45. doi: 10.2188/jea.JE20210220 (PMC10043155; doi:10.2188/jea.JE20210220)
Supplement: Supplementary file 1 [file je-33-236-s001.pdf]

eTable 1. Relationship of coping strategies to all-cause mortality in women after exclusion of participants from a prefectural cancer center: the J-MICC Study, 2004–2014

Coping strategies[a]		*N* Total mortality	Person years	*N* participants	Model 1 HR (95% CI) Follow-up period[b]		Model 2 HR (95% CI) Follow-up period[b]	
					<8.5 years	≥8.5 years	<8.5 years	≥8.5 years
Emotional expression								
	Very few	138	65,826	7,967	Reference		Reference	
	Sometimes	316	216,231	26,025	**0.80 (0.65–0.98)**		0.82 (0.66–1.00)	
	Often/very often	90	66,235	8,141	0.85 (0.65–1.11)		0.84 (0.64–1.11)	
Emotional support-seeking								
	Very few	180	77,462	9,252	Reference		Reference	
	Sometimes	238	172,373	20,704	**0.74 (0.61–0.90)**		**0.77 (0.63–0.94)**	
	Often/very often	125	98,329	12,160	0.85 (0.67–1.07)		0.88 (0.69–1.11)	
Positive reappraisal								
	Very few	52	27,391	3,390	Reference		Reference	
	Sometimes	184	125,835	15,309	0.86 (0.63–1.17)		0.90 (0.66–1.23)	
	Often/very often	308	194,799	23,398	0.90 (0.67–1.21)		0.95 (0.70–1.27)	
Problem solving								
	Very few	69	31,117	3,707	Reference[c]	Reference[d]	Reference[c]	Reference[d]
	Sometimes	219	133,879	16,254	0.85 (0.62–1.16)[c]	1.04 (0.61–1.75)[d]	0.94 (0.68–1.30)[c]	1.09 (0.64–1.85)[d]
	Often/very often	255	182,980	22,132	0.76 (0.56–1.04)[c]	0.84 (0.50–1.40)[d]	0.91 (0.66–1.25)[c]	0.86 (0.51–1.45)[d]

Disengagement						
	Very few	100	48,828	5,810	Reference	Reference
	Sometimes	244	173,612	20,925	0.82 (0.65–1.03)	0.85 (0.67–1.07)
	Often/very often	199	125,183	15,319	0.97 (0.76–1.23)	0.98 (0.77–1.25)

CI, confidence interval; HR, hazard ratio; J-MICC Study, Japan Multi-institutional Collaborative Cohort Study.

[a]Coping strategies were separately included in the models.

[b]Follow-up period was up to 13.9 years. When the proportionality assumption was violated, follow-up period was divided into two phases at the median of 8.5 years.

[c]Follow-up period was <8.5 years (the 0–50th percentile of the total follow-up period).

[d]Follow-up period was ≥8.5 years (the 50–100th percentile of the total follow-up period).

Model 1: Adjusted for age.

Model 2: Adjusted for model 1 + socioeconomic status (educational attainment), physical factors (body mass index, diabetes, hypertension), genetic and environmental factors (father and mother's histories of cancer), behavioral risk factors (smoking status, alcohol drinking, physical activity in daily and in leisure time, sufficiency of sleep), perceived stress level.

Results marked in **bold** indicate a significant association with all-cause mortality ($p<0.05$).

eTable 2. Relationship of coping strategies to all-cause mortality in men after exclusion of participants from a prefectural cancer center: the J-MICC Study, 2004–2014

				Model 1			Model 2		
	N Total mortality	Person years	*N* participants	HR (95% CI)			HR (95% CI)		
				Follow-up period[b]			Follow-up period[b]		
**Coping strategies**[a]				<5.5 years	5.5–8.5 years	≥8.5-years	<5.5 years	5.5–8.5 years	≥8.5-years
Emotional expression									
Very few	239	53,077	6,691	Reference[c]	Reference[d]	Reference[e]	Reference[c]	Reference[d]	Reference[e]
Sometimes	577	155,925	19,169	1.01 (0.79–1.30)[c]	**0.72 (0.56–0.93)**[d]	0.96 (0.72–1.28)[e]	1.05 (0.82–1.36)[c]	**0.82 (0.63–1.07)**[d]	1.00 (0.74–1.35)[e]
Often/very often	165	49,815	6,107	0.90 (0.64–1.26)[c]	**0.71 (0.50–0.99)**[d]	0.99 (0.69–1.41)[e]	0.80 (0.56–1.13)[c]	0.85 (0.60–1.20)[d]	1.04 (0.72–1.51)[e]
Emotional support-seeking									
Very few	580	137,586	16,862		Reference			Reference	
Sometimes	332	98,264	12,227		1.01 (0.88–1.16)			1.05 (0.92–1.20)	
Often/very often	69	22,832	2,858		0.98 (0.76–1.26)			1.05 (0.82–1.35)	
Positive reappraisal									
Very few	161	29,150	3,659	Reference[c]	Reference[d]	Reference[e]	Reference[c]	Reference[d]	Reference[e]
Sometimes	356	95,541	11,808	0.87 (0.64–1.18)[c]	0.81 (0.58–1.11)[d]	**0.60 (0.43–0.84)**[e]	0.82 (0.60–1.13)[c]	0.84 (0.60–1.16)[d]	**0.63 (0.45–0.89)**[e]
Often/very often	461	133,954	16,476	0.83 (0.61–1.12)[c]	**0.66 (0.48–0.91)**[d]	**0.66 (0.48–0.90)**[e]	0.85 (0.62–1.15)[c]	0.75 (0.54–1.04)[d]	**0.70 (0.51–0.97)**[e]
Problem solving									
Very few	135	22,327	2,748	Reference[c]	Reference[d]	Reference[e]	Reference[c]	Reference[d]	Reference[e]
Sometimes	340	85,715	10,554	0.98 (0.71–1.36)[c]	**0.56 (0.41–0.78)**[d]	1.14 (0.75–1.73)[e]	0.98 (0.70–1.37)[c]	**0.65 (0.47–0.91)**[d]	1.23 (0.81–1.88)[e]
Often/very often	505	150,602	18,644	0.80 (0.58–1.10)[c]	**0.54 (0.40–0.73)**[d]	1.10 (0.74–1.65)[e]	0.85 (0.61–1.17)[c]	**0.62 (0.45–0.84)**[d]	1.22 (0.81–1.83)[e]
Disengagement									
Very few	248	54,131	6,571		Reference			Reference	

Sometimes	486	129,017	15,909	0.97 (0.83–1.13)	1.02 (0.87–1.19)
Often/very often	244	75,298	9,437	0.86 (0.72–1.03)	0.90 (0.75–1.08)

CI, confidence interval; HR, hazard ratio; J-MICC Study, Japan Multi-institutional Collaborative Cohort Study.

[a]Coping strategies were separately included in the models.

[b]Follow-up period was up to 13.9 years. When the proportionality assumption was violated, follow-up period was divided into two phases at the median point until the proportionality was confirmed.

[c]Follow-up period was <5.5 years (the 0–25th percentile of the total follow-up period).

[d]Follow-up period was 5.5–8.5 years (the 25–50th percentile of the total follow-up period).

[e]Follow-up period was ≥8.5 years (the 50–100th percentile of the total follow-up period).

Model 1: Adjusted for age.

Model 2: Adjusted for model 1 + socioeconomic status (educational attainment), physical factors (body mass index, diabetes, hypertension), genetic and environmental factors (father and mother's histories of cancer), behavioral risk factors (smoking status, alcohol drinking, physical activity in daily and in leisure time, sufficiency of sleep), perceived stress level.

Results marked in **bold** indicate a significant association with all-cause mortality ($p<0.05$).
